# Experience of Elderly Korean Women with Diabetes and Multimorbidity in Elderly Couple Households: A Qualitative Study

**DOI:** 10.3390/healthcare10091675

**Published:** 2022-09-01

**Authors:** Oksoo Kim, Hyunju Dan

**Affiliations:** 1 College of Nursing, Ewha Womans University, Seoul 03760, Korea; 2 Department of Nursing, Gangdong University, 278, Deahak-gil, Gamgok-myeon, Eumseong-gun, Chungcheongbuk-do 27600, Korea

**Keywords:** elderly women, multimorbidity, lived experience, couple household, phenomenology

## Abstract

Elderly women with multimorbidity in elderly couple households face the double burden of managing their diseases while fulfilling their gender roles. This study aimed to investigate the daily life experiences of elderly women with diabetes and multimorbidity living as part of couple households in Korea. Ten women aged 65 or more with diabetes and multimorbidity and living as part of elderly couple households participated in this phenomenological qualitative study. The data were analyzed with van Manen’s method of study of analytical phenomena. Four essential themes were identified. Participants regarded diabetes and multimorbidity as a part of the aging process and continued to function as caregivers for their husbands and themselves, avoiding becoming a burden to their adult children. The findings of this study could help healthcare providers better understand elderly women with diabetes and multimorbidity and assist in improving the health of such women.

## 1. Introduction

As the elderly population has increased in Korea, the proportion of elderly couple households has reached as high as 48.4% [1]. Therefore, the amount of time that elderly couples spend together also increased [2]. In the past, most elderly Koreans lived with their adult children in the traditional family system and were supported by them [3]. However, the proportion of people who think that it is difficult to expect support from adult children is increasing due to changes in family structure along with modernization [4]. Even though spouses provide mutual support [5], traditional gender role performance, such as helping husbands and doing housework, negatively affects the mental health of older women [6]. The prevalence of chronic diseases such as hypertension, musculoskeletal disease, and depressive disorder among elderly women is higher than that of elderly men [7]. Therefore, older women are more vulnerable to physical and mental health issues than older men; special attention needs to be paid to the daily lives and health care of such women.

According to the International Diabetes Federation (IDF), the global diabetes prevalence in people older than 65 years was estimated to be 20% in 2019 [8]. As of 2018, about three of 10 Korean elderly suffered from diabetes, and the prevalence of diabetes among Korean elderly women was consistently high, 27.5% [7]. Diabetes is a major cause of kidney failure, heart attack, and stroke and requires constant self-care [9]. Among elderly Korean women with diabetes, the incidence of hypertension and hyperlipidemia was 57.2%, higher than that of elderly men at 43.8% [10]. Elderly women with other chronic diseases can experience difficulties in managing these diseases and performing household duties.

The global prevalence of multimorbidity [11], the presence of two or more chronic diseases, ranges from 39.9–76.6% in those over the age of 65 and is 73.0% in Korea, 79.5% for elderly Korean women and 64.1% for elderly Korean men [1,12,13]. Multimorbidity is associated with medication burden [14,15], decreased ability to conduct daily activities [16,17], and decreased quality of life [18]. Multimorbidity management is essential to improving the quality of life by maintaining the health of older adults.

Elderly women with multimorbidity living with their spouses experience the double burden of fulfilling their existing gender roles while managing their diseases. Previous studies have focused on elderly women living alone with a specific single disease rather than women with multiple diseases living with their spouses. Therefore, in this study, we investigated the daily gender role fulfillment and disease management challenges of elderly women with multimorbidity living with their spouses. The results of this study will contribute to improving the understanding of the daily challenges of elderly women with multimorbidity and to improving the quality of life of these women through disease management. The main research question in this study was ‘What are the meanings and essentials of daily life experiences of elderly women with multimorbidity living as part of couple households?’

## 2. Materials and Methods

### 2.1. Study Participants

A phenomenological research method was conducted to explore the lives of elderly women with diabetes and multimorbidity who live as part of an elderly couple. The inclusion criteria for this study were women 65 years of age or older who had been diagnosed with diabetes and multimorbidity of at least one-year duration and who lived only with their spouses. Elderly women who were diagnosed with diseases that affect cognitive function, such as dementia or organic brain disorder, or diseases that limit communicative abilities, such as depression, schizophrenia, and delusional disorders, were excluded from this study. A purposive sampling method was used to select a sample capable of providing vivid and rich experiences to fulfill the purpose of the study. Participants who met the screening criteria were referred by a primary medical institution or welfare institution for the elderly. The researcher met referred subjects in person and explained the purpose and contents of the study. Data were collected from the ten subjects who voluntarily expressed a willingness to participate in the study.

### 2.2. Data Collection

Data collection was performed from March to October 2018. The source of voluntary data was in-depth interviews, one or two face-to-face and 1–3 telephone interviews per participant. The interview location was at the discretion of the participant. To form a rapport for subsequent interviews, the first meeting started with a casual story, creating a comfortable atmosphere. This rapport was maintained through several telephone conversations in addition to the interviews. An interview required 30 to 70 min, and interviews were conducted until the necessary data were saturated. Participant consent was obtained for recording all interviews. Participants’ non-verbal communications, such as facial expressions, behaviors, tone of voice, and silence, were recorded in the field notes. The initial interview questions were open-ended and included: ‘how did you feel when you were diagnosed with several chronic diseases?’, ‘how is your daily life after being diagnosed with several chronic diseases?’, ‘what do you do to manage your several chronic diseases and maintain your health?’, and ‘how is your relationship with your husband?’

### 2.3. Data Analysis

This study was conducted according to the analysis process suggested by van Manen [19] to discover the essential meaning of the daily experiences of elderly women with multimorbidity living only with their spouses. First, the recorded interviews were transcribed verbatim, and the information recorded in the field notes was added to the transcript. The entire transcript was reviewed several times to identify key phrases and statements that captured the basic meaning and significance of the experience. Sentences or words expressing the daily experiences of these elderly women with multimorbidity were identified and displayed, and their meaning and structure were analyzed. The meaning of the experience derived through this process was compared and reviewed with the researcher’s own experience, etymology and idioms, and themes of literary works. To secure the quality of this study, it was evaluated in four aspects: true value, applicability, consistency, and neutrality suggested by Sandelowski [20].

### 2.4. Ethical Considerations

This study was conducted with the approval of the institutional review board (IRB) of the researcher’s affiliated university. Prior to the study, the researcher explained the study’s purpose and content to the participants. Anonymity was assured, as was the participant’s right to discontinue participation without consequences. Voluntary written consent to participate in the study was acquired from those who expressed their intention to participate.

## 3. Results

The demographic and health-related characteristics of participants are presented in Table 1. The average age of the participants was 72.8 years, and the average number of diagnosed diseases was four. The average duration of multimorbidity was 2 to 35 years, and the average number of medications taken per day was nine. The average age of the participant’s husbands was 74.3 years, and the husbands had an average of 1.3 diseases. One husband was classified as ‘healthy.’

As a result of this study, the daily life experiences of elderly women with multimorbidity had four essential themes: ‘treating illness as a part of life and taking care of personal health’, ‘daily functioning while coping with various diseases’, ‘caring for and receiving help from the husband’, and ‘trying not to become a burden to their children’ （Table 2).

### 3.1. Treating Illness as a Part of Life and Taking Care of Personal Health

For the participants, hospital visits were a part of their daily routine, not just an occasional event. Participants acknowledged the need to manage and accept their multiple diagnoses. Participants continued to function while attempting to maintain stable health status. Disease-regulating activities such as diet and exercise tended to be focused on diabetes control. In addition to the current hospital treatment, the participants sought self-education into methods to control their diseases. While medications were an important treatment method, the number of medications required to control their multimorbidity was another burden. The participants usually complied with prescription directives and accepted their diseases as a part of the aging process.

#### 3.1.1. Accepting the Growing Number of Incurable Diseases as a Part of Aging

Symptom relief and the daily routine of managing multiple diseases made disease acceptance second nature for the participants. This acceptance included the incurable status of their diseases and allowed for effective disease management and relief from a negative focus.

*“All the diseases I have are chronic. However, even if the diseases are not cured, I do not get sick often because I take care of myself. So…I have to live with it (diseases) like friends.”*

(Participant A)

Hospital visits, like visits with friends, were a part of the participants’ daily routines. The participants’ deteriorating health status was rationalized and accepted as part of the aging process as participants commiserated with other sick individuals in their age group.

*“My husband and I both go to the hospital often. I went to the hospital for treatment this morning... Going to the hospital is the most important part of our daily routine.”*

(Participant B)

*“I think the older I get, the more diseases I get. I think a lot of people at my age have knee pain. I am 71 years old, and this year I feel that my energy is lower than last year. So, I feel I’m getting older.”*

(Participant C)

#### 3.1.2. While Facing Multimorbidity, Focusing on Managing Diabetes to Include Complementary Treatment Methods Obtained through Self-Education

Diet management was the participants’ first concern, and managing diabetes was the priority among the multimorbidity. Dietary management was, however, difficult because of sharing meals with their husbands. Managing diabetes through exercise was also important to the participants. However, some participants were affected by arthritis; this limited exercise options.

*“My husband eats salty food so much. I keep trying (to be careful with salty food), but it doesn’t work. Even if I ask him to eat blander food, he always says (if the food is not salty enough) that it doesn’t* *taste good. I cook food for my husband separately and add salt to it, but I also end up eating salt (laughs). Even though I’m more careful than before…it is not easy.”*

(Participant E)

Participants wanted and sought more treatment options than their physicians provided. Some dietary and medicinal therapies were obtained through self-education methods. These therapies were tried and assessed for effectiveness.

*“I took painkillers for back pain, but I saw on TV that it was not good to take too many painkillers. So I stopped taking the medicine and (my back) hurts a lot. (Omission) I don’t eat red ginseng because my doctor told me not to eat it. I don’t take anything else (except the medicine), but these days I don’t have energy, so I should have to eat something like red ginseng…”*

(Participant F)

#### 3.1.3. Following Medication Regimens despite Difficulties Associated with Managing Multimorbidity

The sheer number of medications required to control their multimorbidity was a source of participant distress. Participants understood the necessity of the medications and the disease for which each medication was used but were not well-informed on the mechanism of action and side effects of the medicines. Medication changes were common with changes in symptoms, but participants did not always know which medication had been changed despite easy access to this information.

*“A high blood pressure medicine, a hyperlipidemia medicine, and a medicine that prevents dementia, *etc.* (I take) a handful. (Omission) Ha (Sighs)… Anyway, it’s hard because I take several medications.”*

(Participant D)

*“My blood pressure has gone down, so I went to the doctor and changed the medicine again. My blood sugar level was slightly higher this time... But I don’t think I’ve changed my diabetes medication...ah… no, no, it seems that even one pill for diabetes has changed...ah…I do not know for sure.”*

(Participant B)

Participants sometimes forget to take their medication on time because the time varies depending on the medication. Therefore, they tried to find their own way not to forget the time to take their medication. Some participants did not follow prescription guidelines because of over-treatment fears.

*“There are separate medicines for diabetes and rheumatoid arthritis, but I take them (both medicines) together, so I don’t forget to take them. I don’t know if it’s okay to take them together, but the doctor told me to take both medicines 30 minutes after a meal, so I take them together. However, I stopped taking the rheumatoid medication that I used to take once a week. (Why did you stop?) I thought it would be bad if I kept taking the medicine even though I wasn’t sick.”*

(Participant G)

### 3.2. Daily Functioning While Coping with Various Diseases

All of the participants continued to function as before the multiple diagnoses. With the departure of children from the household, household size decreased. However, the household chore requirements of the remaining couple were unchanged. While social functions and responsibilities decreased, self-education efforts continued. The participants continued to support their children through activities such as food preparation and childcare. However, some participants with degenerative musculoskeletal disorders complained of difficulties associated with chronic pain; for the study participants, freedom of movement was vital to maintaining normal functioning.

#### 3.2.1. Continuing Household Upkeep and Social Activity Participation despite Illnesses

Even though the participants had multimorbidity, effective disease management prevented dramatic changes in daily functional abilities and limited the need for outside assistance. All of the participants’ children were married, so the household size was only a couple. Participants rested when necessary, dividing the workload between frequent breaks. Although the size and frequency of meetings and outside activities decreased, participants were engaged in external activities such as learning foreign languages, exercising, and participating in classes.

*“I live a busy life because I have to go to the hospital for my hurt back, to exercise, and to go to senior classes. I have also to do housekeeping. So I am busy.”*

(Participant F)

Painful diseases presented the greatest challenges. Injections or physical therapy were provided to control the pain caused by musculoskeletal diseases such as arthritis or intervertebral disc herniation, but the therapeutic effects were temporary. Limited activity and pain endurance were standard for those with these conditions.

*“Back pain is the hardest for me. I can’t walk freely, and if I walk a lot, my back hurts more, so I have to sit and rest a few times… I don’t think diabetes or high blood pressure is too difficult because all I need to do is take medicine. But the back pain gets better only after taking medication or physical therapy and comes back after a while... It’s hard, but I just live with it.”*

(Participant D)

#### 3.2.2. Helping Children, Such as Cooking for their Children or Caring for Their Grandchildren

The participants did not expect their children’s help and wanted to provide help to their children despite their multimorbidity. The participants empathized with their children’s situations and felt that helping their children was a responsibility.

*“Our youngest son is part of a dual-income couple, so my husband and I are taking care of our grandchildren. If we don’t help, it’s difficult for them (the youngest son and his wife) to live. (Shakes head) (If we don’t help them) they have to leave the baby with someone else… I think it’s bad. I think it’s normal as a parent (to be helping children).”*

(Participant C)

### 3.3. Caring for and Receiving Help from the Husband

The participant’s feelings toward their children’s departure from the household were varied. There were feelings of sadness and emptiness, but, at the same time, the lower workload brought feelings of happiness. The participants and their husbands had shared difficulties and pleasures for several decades; the difficulties occasionally engendered a degree of antipathy. Participants also stated that there was cooperation and empathy with their spouses. Their husbands’ health and being a caregiver were important to the participants. Participants were optimistic when considering life after the death of their husbands but felt that the absence would negatively affect their own health.

#### 3.3.1. Being Husband’s Caregiver While Coping with Multimorbidity

The participants were caregivers to both themselves and their husbands; their husbands mostly had chronic diseases. A major source of concern for the women was their diet. Fulfilling the husband’s favorite food desires while trying to manage their own diabetes was a significant concern. The husband’s alcohol consumption and tobacco use were also a source of concern, as was the potential deterioration of the husband’s health.

*“My husband also has diabetes, so I was careful not to increase our glycemic index, so he said that food does not raise his blood sugar. But when my husband drinks 6–7 cups of instant coffee a day, his blood sugar rises dramatically... So these days, I don’t buy mixed coffee and I tell my husband to drink black coffee, so (he) drinks black coffee at home, but I think he’ll drink mixed coffee outside.”*

(Participant I)

#### 3.3.2. Living with Compassion and Consideration for Husband’s Aging

The participants were generally considerate of their husband’s bouts with illness and aging; the participants chose to live in a state of cooperation with their husbands. The presence of their husband increased their willingness to care for themselves.

*“Wouldn’t my health be bad if I lived alone? If I lived alone, I’d probably be lazy. If my husband lives with me, at least I’ll prepare a meal. But I won’t eat well if I’m alone because I don’t enjoy it.”*

(Participant H)

#### 3.3.3. Receiving Husband’s Help for Some of the Housework

The participants took for granted that they were primarily responsible for ensuring that housework was accomplished. Although there were differences depending on the husbands’ health conditions, most participants said that the husbands helped more now than in the past. Some participants did not feel that the husband was motivated to help because of their wife’s comorbid health issues.

*“My husband washes his dishes by himself. He also runs the vacuum every now and then. When my husband was young, he didn’t do (housework). He didn’t even come into the kitchen.”*

(Participant H)

### 3.4. Trying Not to Become a Burden to Their Children

The participants were driven by a strong desire to help, not hinder, their children. Participants did not expect to be supported by their children despite the presence of multimorbidity and did not want to be a source of concern for their children. Therefore, the participants avoided discussions of economic difficulties and contact with their children when they were actively sick. Participants were concerned that their old age would be a burden on their families, especially their children. Participants wanted to live independently without major problems but were afraid that their health would worsen and cause suffering. Their goal was to maintain their health status, such as not to impose upon their children.

#### 3.4.1. Avoiding Conversations concerning Pain and Discomfort to Keep Children from Worrying

The participants said that because their children are busy with their own lives and are not in a situation to help their parents, conversations about symptoms, illnesses, and financial difficulties are avoided to keep the children free from the burden. The participants also expressed regret over asking their children for favors. Most of the participants responded that medical expenses increased as the number of diseases increased but that Korea’s health insurance was usually enough to pay the medical bills.

*“After I started getting arthritis, my kids told me not to work. But I tell them that I’m OK even if I’m sick. (Laughs) There’s no reason to make the children worry about me. Illness is something I have to manage. Just because my children know doesn’t mean my illness will be cured, it just puts a burden on them.”*

(Participant G)

#### 3.4.2. Not Expecting to Receive Support from Children and Seeking Solace from Within Occasionally

Participants were not willing to ask for help from their children despite taking care of their own elderly parents in the past when necessary. Social perceptions have changed; when the participants were younger, parental care for the elderly was expected. Now the participants did not expect their own children to house and care for them. Another consideration was that the participants did not want to “walk on eggshells” while living with their children. Discussion of feelings of disappointment with children was avoided; the participants focused on their own, not their children’s behavior.

*“Everyone is upset because of their children and husband sometimes. In the past, when I was concerned about my children and husband (especially about upsetting things), my body would hurt. Now… I live without worrying about them. I have to be satisfied because all my kids are healthy.”*

(Participant J)

#### 3.4.3. Hoping Not to Be a Burden on the Children Due to Worsening Health

Participants were worried that their health would deteriorate in the future and that they would become a burden to their children. In addition, the participants wanted to avoid suffering and hoped for a pain-free death.

*“My wish is to die without suffering. (Her eyes began to well up) I’m afraid my kids will suffer because I’ve been lying in bed for a long time… That’s what worries me the most.”*

(Participant B)

## 4. Discussion

The purpose of this study was to investigate the daily life experiences of elderly women with diabetes and multimorbidity living as part of couple households in Korea. Participants in this study were accustomed to living with multiple incurable morbidities. Our results are consistent with the findings of Duguay, Gallagher, & Fortin [21] that patients with multiple chronic diseases accept the diseases as a part of their daily lives and adapt to the disease.

Patients with multimorbidity might have limited social lives due to physical problems [21,22]. However, the participants in this study continued to engage in social activities such as education programs for the elderly and social welfare center programs. Because participants with coronary artery disease or respiratory disease were not included in this study, few restrictions on social activities were expected. In this study, some participants with musculoskeletal disorders endured the associated pain and continued to function. Pain and arthritis are factors that limit daily functioning and lower the quality of life [21,23]. Therefore, further studies that consider the severity of the diseases and the effects on the ability to perform daily activities are needed.

Although the participants of this study had multimorbidity, their health management focused on diabetes. This might be the result of the emphasis on lifestyle modifications in managing diabetic symptoms. However, some other chronic diseases also respond to lifestyle changes; for example, diet control and exercise can also help improve hypertension and hyperlipidemia. Therefore, healthcare providers need to develop multidisciplinary programs for patients with multimorbidity that are tailored to individual characteristics.

Rosbach and Andersen [15] reported that patients with multimorbidity had difficulty in disease management due to varying medical recommendations for individual diseases. The participants in the present study were managed for several diseases by one doctor at one hospital; inconvenience associated with multiple doctors at multiple facilities was avoided. Medication is important in the treatment of multimorbidity, but as the number of diseases increases, the type and number of medicines increase. Problems with medication non-compliance, misuse, and abuse arise [14,24]. As the number of diseases increased, the participants felt burdened by the increase in the number of medications. Prescription non-compliance resulted from participant lapses in memory or from their concerns about the effects of long-term medicine use. For the elderly with multimorbidity, effective measures for maintaining a regular dosage schedule need to be implemented. In addition, to ensure that medicines are taken as prescribed, repeated interventions might be needed to improve adherence [25]. Regular education programs on the effects and side effects of medications are also required.

Most of the participants’ husbands also suffered from various diseases, but there were no problems with cohabitation because the frequency of difficulties was low. However, participants were concerned about their husbands’ lack of self-care. The husbands’ alcohol consumption and tobacco use were of particular concern. Most stated that the health status of their husbands did not affect the lives of the participants. However, one participant who was reluctant to reveal her husband’s diagnosis stated that her husband’s illness affected her life. These results were partially consistent with previous studies [26] that reported that the husband’s chronic disease was one of the factors influencing the wife’s life satisfaction. According to the results of previous studies, the burden for caregivers of those with severe chronic diseases such as dementia or stroke affects the quality of life of caregivers [27,28]. Therefore, we assumed that the burden of support for spouses of elderly women with multimorbidity would be greater than that of healthy women. Therefore, a study on the effects of spousal chronic disease severity is required, particularly in those elderly women with multimorbidity.

Participants in this study controlled their diets to manage their diseases; these dietary controls were influenced by their husbands. Fulfilling the spouse’s desire for certain foods might have affected the participant’s ability to provide themselves with an optimal diet for their medical conditions. Alternatively, a couple’s lifestyle influences both people [29]; since most of the spouses of the participants also had chronic diseases, changes in diet were necessary for the spouse as well. However, elderly people with diabetes need strict dietary control to control blood glucose levels and prevent complications. Therefore, when healthcare providers provide dietary counseling to elderly women with diabetes, it is necessary to provide specific examples of diet in consideration of the health status of their spouses. Exercise, as well as diet, is crucial for diabetics in controlling blood glucose. However, while the elderly women who participated in this study were well aware of the importance of diet, they were insufficiently aware of that of exercise. According to a previous study, only 28% of elderly Korean women with diabetes performed regular walking exercises [10]. Therefore, it is necessary for healthcare providers to include education on exercise when providing dietary education to elderly women with diabetes.

Participants’ ideas reflected societal changes. The support that the participants provided to their own elderly parents was no longer expected of their children. Some participants were helping with household chores for married adult children or caring for their grandchildren and were concerned that, if their health deteriorated, the support provided to their children would no longer be available. Most Korean elderly women believe in helping their married adult children with housework or childcare. Regardless of gender or disease, individuals wish to finish their lives without pain and without burdening others [30,31,32]. Healthcare providers need to operate chronic disease management programs for elderly women with diabetes and multimorbidity by considering the health of their spouses, which may affect the health of elderly women.

### Strengths and Limitations

The strength of this study was its confirmation of the essence of daily life experiences of elderly women with multimorbidity who live only with their spouses. Because qualitative research conducted through in-depth interviews with elderly women with multimorbidity was lacking, the results of this study could be utilized to improve the health and quality of life of such women.

This study has limitations. First, the participants of this study were able to engage in outdoor activities and did not experience any serious complications. Therefore, there is a limit to the generalizability of our results. Second, the participants lived in a metropolitan area, all but one owned their homes, and all had sufficient income that prevented the medical expense burden for multimorbidity management. Therefore, the findings might not be generalizable to low-income elderly women and their spouses.

## 5. Conclusions

In this study, elderly women living only with their spouses considered their diabetes and multimorbidity to be a part of the aging process and continued to function in their daily lives. These elderly women were their husbands’ caregivers and attempted to avoid becoming a burden to their adult children. The results of this study can be used by healthcare providers to understand and improve the health of elderly women with diabetes and multimorbidity.

## Figures and Tables

**Table 1:** General and health-related characteristics of study participants.

Participants	Age (yrs)	Education Level	Diagnosis	Diagnosis Period (yrs)	Number of Daily Medications	Age of Husband (yrs)	Diagnosis of Husband
A	74	Middle school	Osteoarthritis	Over 25	0 (Under treatment with acupuncture)	74	Pancreatic cancer
			Cerebrovascular disease	18			
			Arrhythmia	15			
			Diabetes	2			
B	79	High school	Diabetes	25	5	87	No disclosure of her husband’s diagnosis
			Hypertension	Over 10			
			Osteoarthritis	Over 10			
C	70	High school	Diabetes	11	6	73	Diabetes Hyperlipidemia
			Hyperlipidemia	Unknown			
			Hypertension	Under 10			
			Osteoarthritis	2–3			
			Osteoporosis	2–3			
			Hypothyroidism	5 months			
			Colorectal cancer	2			
D	80	Under elementary school	Hypertension	Over 40	12	78	Hypertension Herniation of lumbar disk
			Diabetes	35			
			Hyperlipidemia	Unknown			
			Thyroid cancer	Over 20			
			Herniation of lumbar disk	3			
			Lumbar spinal stenosis	7 months			
E	71	High school	Chronic hepatitis	12	5.5	72	Diabetes Hyperlipidemia
			Diabetes	10			
			Hypertension	9			
			Osteoporosis	2			
			Hyperlipidemia	2			
			Osteoarthritis	7			
F	79	Under elementary school	Diabetes	15	5	82	Hypertension Back pain
			Hypertension	Over 10			
			Lumbar spinal stenosis	1			
G	65	High school	Diabetes	20	8	67	None
			Rheumatoid arthritis	2			
H	72	High school	Diabetes	30	8	72	Diabetes
			Hypertension	10			
			Panic disorder	1			
I	65	High school	Breast cancer	7	7	68	Diabetes
			Diabetes	7			
			Osteoporosis	7			
J	73	Under elementary school	Hypertension	20	8.5	70	Hypertension
			Diabetes	10			
			Osteoarthritis	1			

**Table 2:** Essential themes of daily life experiences of Korean elderly women with multimorbidity.

Theme	Essential Theme
Accepting the growing number of incurable diseases as a part of aging	Treating illness as a part of life and taking care of personal health
While facing multimorbidity, focusing on managing diabetes to include complementary treatment methods obtained through self-education	
Following medication regimens despite difficulties associated with managing multimorbidity	
Continuing household upkeep and social activity participation despite illnesses	Daily functioning while coping with various diseases
Helping children, such as cooking for their children or caring for their grandchildren	
Being husband’s caregiver while coping with multimorbidity	Caring for and receiving help from the husband
Living with compassion and consideration for husband’s aging	
Receiving husband’s help for some of the housework	
Avoiding conversations concerning pain and discomfort to keep children from worrying	Trying not to become a burden to their children
Not expecting to receive support from children and seeking solace from within occasionally	
Hoping not to be a burden on the children due to worsening health	

## Data Availability

Not applicable.
